# Genetic Instability of a Polydactyl Hypopigmented Cat With Squamous Cell Carcinoma—A Case Report

**DOI:** 10.3389/fvets.2020.00258

**Published:** 2020-05-12

**Authors:** María Evarista Arellano-García, Marian Eliza Izaguirre-Pérez, Leonardo Daniel Molina-Noyola, Idalia Yazmín Castañeda-Yslas, Roberto Luna-Vázquez-Gómez, Olivia Torres-Bugarín

**Affiliations:** ^1^Laboratorio de Genotoxicología Ambiental, Facultad de Ciencias, Universidad Autónoma de Baja California, Ensenada, Mexico; ^2^Facultad de Medicina, Universidad Autónoma de Guadalajara, Zapopan, Mexico; ^3^Programa Internacional de Medicina, Universidad Autónoma de Guadalajara, Zapopan, Mexico

**Keywords:** albinism, hypopigmented, polydactyl, genomic instability, micronucleated erythrocytes, squamous cell carcinoma

## Abstract

Polydactyly, hypopigmentation, and squamous cell carcinoma are common in cats. However, a cat exhibiting all of these conditions has not yet been reported. This study presents the case of a 14- year-old male Mexican cat, hypopigmented, with supernumerary fingers, two preaxial and one on each posterior limb, admitted to the clinic with a lesion in the left periocular region. The cat was subjected to a general physical examination, blood, and urine chemistry, as well as a biopsy and genomic instability assessment with an analysis of the red blood cells (RBC) micronucleated erythrocytes (RBC-MNE) in the peripheral blood. The biopsy was positive for squamous cell carcinoma, and the RBC-MNE count (8.6 MNE/1000 erythrocytes) was high compared to that previously described in other domestic cats or wild cats. Thus, the genomic instability of the RBC-MNE could be used as an indicator to identify clinical conditions of felines, particularly those with one of the characteristics exhibited by this Mexican cat. The RBC-MNE test is the most widely used in the world for the evaluation of DNA damage, but to our knowledge, it has not been used to identify vulnerable non-human specimens.

## Introduction

Genomic stability is essential to guarantee the health and perpetuity of the species; however, the dynamic nature of DNA allows for modifications in structure (mutations) due to endogenous (replication, recombination, repair, or reactive metabolites) or exogenous events (chemical, physical, or biological). Any form of genotoxic stress due to internal or external factors leads to the formation of micronuclei, which serves as an indicator of chromosomal instability. Chromosomal damage and micronucleus formation play a significant role in the pathogenesis of diverse malignancies. Genomic instability can manifest itself from the molecular level passing through structural damage or the malfunction of organs and tissues to situations that jeopardize the life of the individual. Several studies have shown that the micronucleus assay may be used as a risk prediction, screening, diagnosis, and prognosis tool, as well as an indicator for the cancer treatment response (1–3). This assay is widely accepted, versatile, and uses samples from the bone marrow or the peripheral blood erythrocytes (4). Despite the versatility and robustness of the micronucleus test established in humans, in animals, this study is focused as an end-point biomarker used for laboratory animals or environmental monitoring, not for the identification of organisms vulnerable to genetic damage or instability (5, 6).

**Figure 1:**
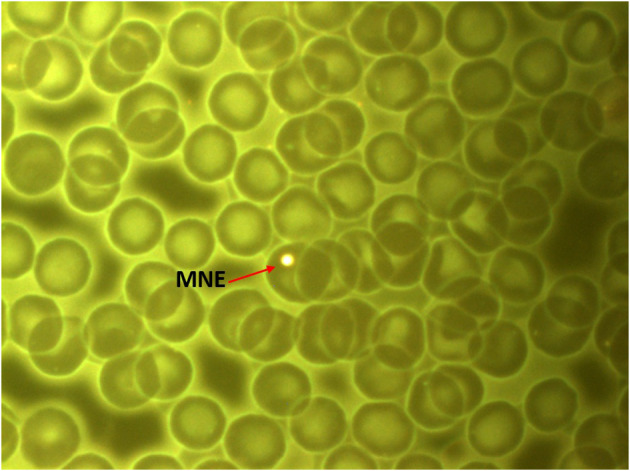
Peripheral blood smear of the subject cat (*Felis catus domesticus*). Red arrow: Micronucleated erythrocyte. Stain Fluorescence IVFL filter 450–490 nanometers. Axiostar Plus microscope. Planachromatic objective 100x/1.25 oil. Axiocam camera 1cc. 1000 real increases. Photo courtesy Dr. Olivia Torres Bugarín, Evaluación de Genotoxicos Laboratory, Programa Internacional de Medicina UAG.

Micronuclei were first discovered at the end of the nineteenth century by Howell and Jolly, who found small inclusions in the blood taken from cats and rats (4). In 1996, the frequency of RBC-MNE in healthy adult cats was initially used as a biomarker (7). A subsequent experiment was performed in young cats (6 to 8 weeks old), where RBC-MNE was induced using micronucleogenic agents colchicine and arabinose C., which confirmed the high sensitivity of cats to genotoxic damage and genomic instability (8). Today, micronuclei are currently referred to as Howell-Jolly bodies (Figure 1) and are formed in the bone marrow. In humans, the spleen removes the abnormal or older cells, and erythrocytes with micronuclei inclusions; however, the spleen of felines is not as efficient at filtering micronuclei as other mammals (6, 9). Thus, Howell-Jolly bodies found in felines could allow for the use of the micronucleus assay as a diagnostic tool.

Albinism (*albus* = white) is a heterogeneous genetic disorder in which the most frequent inheritance pattern is autosomal recessive, resulting in a decrease or total absence of skin pigmentation (10). The genetic material of albinos is subject to damage by oxidative stress and solar radiation, leading to a high predisposition to cancer (11). Two forms of mutant albinism are known in mammalian genetics. The first is complete albinism, in which pigmentation is lacking in the hair, skin, or deeper tissues of the body resulting in a pink-eyed animal with white hair. The second is acromelanic albinism with variable amounts of pigment in the extremities (nose, ears, feet, and tail), eyes are often pink, ruby, or blue (12).

Polydactyly is autosomal dominant and the most common hereditary malformation of the limbs, heterogeneously manifesting itself within many types of vertebrates (13), as an isolated trait, or related to a syndrome (14). The affected individuals are born with supernumerary fingers in one or several extremities, where a great intrafamilial and interfamilial clinical variability exists. Aside from the increase in the number of digits, the carpus and tarsus conformation can also be altered. Cats usually have 18 toes, 10 on the front limbs, and 8 on the hind legs, without clinical or functional adverse effects caused by polydactyly. However, polydactyly can be associated with other anomalies such as “hamburger feet” or “mitten paw.” In the former, a well-differentiated supernumerary finger appears next to the fifth finger (preaxial), meanwhile, for the latter, a less common anomaly, the malformed supernumerary finger appears next to the first finger (postaxial) (13–15).

## Case Report

**Figure 2:**
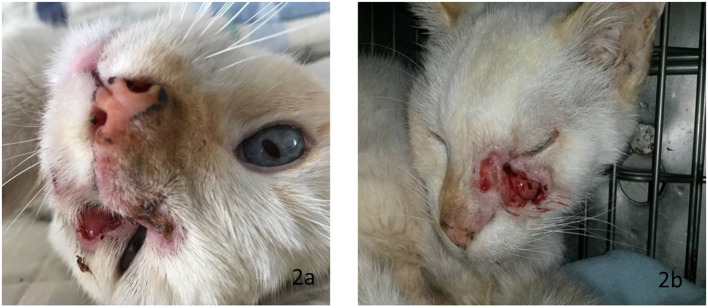
**(a)** Eyes: light blue, large nut-shaped, separated, slightly oblique. **(b)** Fistula in the left maxilla with secretion and foul odor.

**Figure 3:**
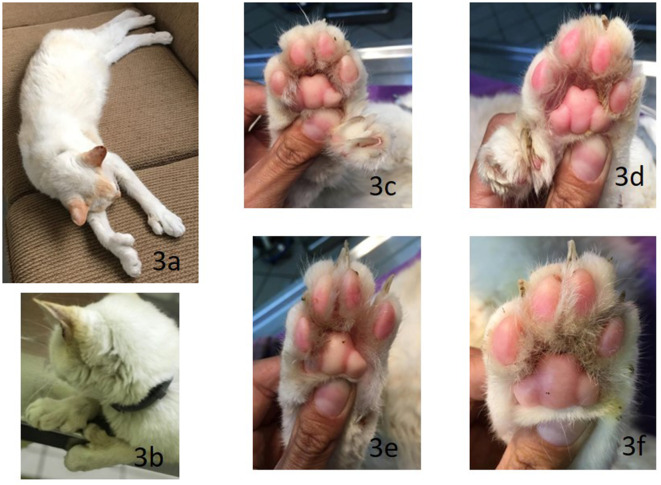
14-year-old neutered Mexican male cat. **(a,b)** Head: in an equilateral triangle. Paws: short, of medium bone, strong musculature, medium-sized feet, round, and compact. Polydactyly in the four extremities, **(c,d)** two accessory fingers preaxially in front and **(e,f)** one in the back.

Here we present the case of a domestic 14-year-old male Mexican cat with short hair, white coat, and blue eyes, with a marked photophobic behavior (Figures 2, 3). The cat was adopted from another family, neutered, and always lived inside. The owner signed an informed consent form to participate the laboratory tests and to use the results in a scientific article. The clinical features suggest an acromelanic albinism, but it was not possible to genotype this animal. Polydactyly was present in the extremities, two supernumerary digits in the front limbs, and one in the hind legs. The general physical examination showed a body temperature of 39.5°C, actinic keratosis, an abundance of ectoparasites, diarrheal episode, followed by asthenia and adynamia (weakness), and a fistula in the left maxilla at one year of develop.

## Diagnostic Assessment

### Laboratory Tests

The blood chemistry (Table 1), reported as part of a routine complete blood count (CBC) mild eosinophilia, and alteration in bands, as well as in concentration of glucose, urea, cholesterol, and amylase, consistent with the presence of ectoparasites. Also reported hyperamylsemia, hyperchloremic, probably due to catabolism or muscle damage which is consistent with the low body mass index, aging, and hypoalbuminemia due to lack of nutritional intake.

**Table 1:** Hemogram, urinalysis and complete laboratory profile.

**Date**	**Analyte**	**Units**			**Results**
		**Jun 2015**	**Oct 2016**	**Oct 2017**	
Hematocrit	L/L	0.30	0.32	0.34	0.24-0.45
Hemoglobin	g L	99	105	106	80-150
Erythrocytes	X 1012/L	6.4	6.5	6.7	5-10
VGM calculated	fL	47	49	46	39-55
CGMH calculated	g/L	330	328	341	300-360
Reticulocytes	X 109/L	–	–	—	<60
Platelets	X 109	340	352	338	300-700
Total solids	g/L	68	70	76	60-81
Leukocytes	X 10 9/L	9.7	11.1	9.1	5.5-19.5
Neutrophils	X 10 9/L	7.6	5.5	5.4	2.5-12.5
Bands	X 10 9/L	0	1.4^*^	0.1	0-0.3
Metamyelocytes	X 10 9/L	0	0	0	0
Myelocytes	X 10 9/L	0	0	0	0
Lymphocytes	X 10 9/L	1.4	1.9	2.7	1.5-7.0
Monocytes	X 10 9/L	0	0.2	0.1	0-0.8
Eosinophils	X 10 9/L	1.7^*^	2.3^*^	0.8	0-0.9
Basophils	X 10 9/L	0	0	0	0
Glucose	mmol/L	4.0	7.8	8.2^*^	3.8-7.9
Urea	mmol/L	10.2	12.3^*^	33.9^*^	4.1-10.8
Creatinine	μmol/L	73	60	106	56-176
Cholesterol	mmol/L	3.03	3.74	5.81^*^	1.78-3.87
Triglycerides	mmol/L	0.3	0.2	0.2	0.3-0.9
Total bilirubin	μmol/L	2.6	1.7	1.1	<6.8
Conjugated bilirubin	μmol/L	1.9	1.2	0.8	–
Unconjugated bilirubin	μmol/L	0.7	0.5	0.3	–
Alaninamino transferase	U/L	54	64	64	<72
Aspartateamino transferase	U/L	42	34	42	<61
Alkaline Phosphatase	U/L	33	44	94	<107
GGT	U/L	7	5	7	<10
Amylase	U/L	4741^*^	2984^*^	3275^*^	<1800
Creatine kinase	U/L	395^*^	849^*^	455^*^	<277
Total proteins	g/L	64	65	63	59-81
Albumin	g/L	28	25^*^	25^*^	26-38
Globulins calculated	g/L	36	40	38	29-47
A/G ratio calculated	–	0.78	0.62	0.65	0.58-1.16
Total calcium	mmol/L	2.39	2.03	2.63	2.05-2.76
Phosphorus	mmol/L	1.27	1.26	1.98^*^	0.96-1.96
Potassium	mmol/L	4.7	4.5	4.4	3.6-5.3
Sodium	mmol/L	154	156	152	143-158
Chlorine	mmol/L	121^*^	128^*^	122^*^	111
Bicarbonate	mmol/L	18	15	22	14-24
Anion gap calculated	mmol/L	20	18	21	10-27
Osmolality calculated	mOsm/kg	317	319	329	290-330
Difference of strong ions calculated	mmol/L	33	28	30	30-40
**Urinalysis (chemical test)**					
pH		6	6.5	6	
Protein	g/L	0.3	1.0	0.15	
Glucose	mmol/L	0	0	0	
Ketones		Normal	Normal	Normal	
Bilirubin		Negative	Negative	Negative	
Blood		1+	2+	Negative	

* *Outside of reference values*.

### Histopathology Report

**Figure 4:**
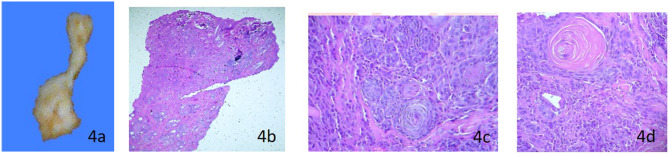
Fistula histopathological analysis macroscopic description: **(a)** Left periocular skin (1.2 × 0.8 cm), homogeneous white surface, broad apical end interspersed with adipose tissue, and irregular edges. Microscopic description **(b)** Epidermis with loss of continuity of the epithelium, partially covered by serocellular scabs. Below, poorly demarcated and infiltrating neoplastic flat stratified epithelial tissue arranged in interconnected islands and trabeculae. **(c)** Pleomorphic cells with hypereosinophilic cytoplasm with small vacuoles; one or two prominent, pleomorphic nuclei with fine granular chromatin; 7 atypical mitoses/10 randomized fields (400X), anisocytosis and anisokaryosis. **(d)** Marked desmoplasia, and some nests, the neoplastic cells (also present in surgical border) are organized around concentric sheets of keratin (keratin beads). Diagnosis- Squamous cell carcinoma well-differentiated proliferative cells, generalized moderate ulcerative epidermitis.

The cytology showed chronic inflammation, few bacteria, and abundant anucleate squamous cells. An incisional biopsy showed the presence of a well-differentiated squamous cell carcinoma (Figure 4), associated with an ulcerative epidermis in the periocular region (Figure 2).

### Genomic Instability Probe

Micronucleus assay quantified 8.6 MNE/1,000 erythrocytes. This test used a drop of peripheral blood, a smear was made on a pre-cleaned microscope slide, fixed, and then stained with acridine orange supravital stain. The frequency of RBC-MNE was examined under the microscope (Carl Zeiss IVFL Axiostar Plus® equipped with fluorescence IVFL filters of 450–490 nm) at 100× optical amplification (planachromatic, 1.25 oil). RBC-MNE quantification was performed using 10,000 erythrocytes as previously reported, but the result was reported in 1,000 erythrocytes (7, 8, 16).

### Therapeutic Intervention, Follow-Up, and Outcome

Chemotherapy treatment was recommended; however, the owner preferred a palliative treatment consisting of hygienic dietary measures and medications such as prednisone, enrofloxacin, ranitidine, intravenous hydration, antibiotics, analgesia, and a renal support diet. The patient died a year later due to health complications.

## Discussion

While both polydactyly and albinism are common in domestic cats, the presence of both mutations in same cat is not a common finding due to dissimilar inheritance patterns (17). However, quantification of a remarkable amount of erythrocytes (8.6 MNE/1000) was likely the result of high genomic instability that could be related to the presence of both acromelanic albinism and the squamous cell carcinoma. As seen in Table 2 the RBC-MNE frequency found in this cat was 100-fold higher than the average value found in domestic healthy adult cats or wild felines (0.84 /1000 erythrocytes), and 2- to 3-times higher than the frequency found in kittens (6–8 weeks-old) exposed to micronucleogenic agents (7, 8, 16).

**Table 2:** Micronucleus frequency in the feline peripheral blood.

**Age**	**Feline species**	***n***	**MNE 1,000 erythrocytes**		**References**
			**Mean**	**Min-Max**	
Adults	Albino cat, polydactyly and carcinoma	1	*8.6*		Our data
	Domestic cat *Felis domesticus*	6	0.84	0.6–1.3	(7, 16)
	Siamese cat (*Felis domesticus)*	3	1.1	1.0–1.1	
	Lion (*Panthera leo)*	5	0.4	0.55–0.8	
	Yaguaroundi (*Felis yagoaroundi)*	1	0.45	0.45	
	*Lynx (Felis ruffus)*	3	0.08	0.05–1.95	
	*Ocelote (Felis pardalis)*	1	1.35	1.35	
**Micronucleated erythrocytes in cats treated with colchicine** **+** **Ara-C**					
**Age**	**Domestic cat**	***n***	**MNE/1,000**		**Reference**
			**24 h**	**96 h**	
6-8 weeks	Control	10	2.3 ± 1.1	2.2 ± 0.9	
	Colchicine^*^+ Arabinoside-C^*^	10	2.4 ± 0.7	3.4 ± 1.2	(7)

*n, Sample size; Min-Max, Minimum-Maximum; MNE, Micronucleated erythrocytes (biological marker of genotoxic damage and genomic instability); Colchicine (6mg/kg) + Arabinoside (0.26 mg/kg)*,

* *daily intraperitoneal*.

### Albinism and Genomic Instability

Melanin, a complex polymer, is the most prevalent pigment in nature and associated with providing color to plants, animals, and microorganisms. This polymer could inactivate both Reactive oxygen species and free radicals. Melanin has been found to sequester redox metals and toxic organic compounds, act as a barrier against microorganisms, and contributes to immunological protection against environmental stress and solar radiation. This polymer is considered a very efficient photoprotector, capable of absorbing the entire visible and ultraviolet spectrum. Melanin transforms energy into harmless heat and prevents indirect damage to DNA; therefore, lack of melanin, (albino organisms), leads to a higher risk of skin cancer (11, 18, 19). The intrinsic and extrinsic regulation mechanisms of pigmentation are complex and have not yet been completely elucidated (19–21); however, hypopigmentation in albinism is caused by a mutation that could block the metabolic pathway of phenylalanine from dihydroxy phenylalanine (DOPA) to melanin (21). Complete albinism in cats is often associated with a mutation in the tyrosinase gene (TYR) (22). Patients with decreased pigmentation (vitiligo or albinism) had a higher frequency of micronucleated cells compared to that of healthy people (23, 24). This fact agreed with the genetic instability and carcinogenesis induction observed in an organism without the protection from radiation or the neutralization of free radicals performed by melanin (11, 18). Moreover, in albino cats, the risk of metastasis is 13.4 times greater compared with non-albino cats, and this risk increased if the cat lived in an area with high solar radiation (25). All of these facts likely contributed to the development of the squamous cell carcinoma within the subject cat. This cancer is a malignant, locally infiltrating, slow-growing neoplasm whose surgical removal produced a high recurrence rate that depended on whether the surgery was complete or incomplete and whose potential for metastasis, depended on the proximity to the lymph nodes. In this case report, genomic instability produced by lack of protective melanin combined with the development of a squamous cell carcinoma could be the reason for the very high RBC-MNE frequency observed.

### Cancer and Genomic Instability

Genetic instability plays an important role in carcinogenesis; however, the mechanisms involved have not yet been fully elucidated. The tumor cell genome is more susceptible to chromosomal alterations such as breaks, exchange of sister chromatids, and changes in ploidy than the normal cell genome, suggesting that these alterations may be responsible for the development of tumors (26). In this case, it was not possible to identify if the genomic instability observed was the cause or the consequence of the hypopigmentation and squamous cell carcinoma. However, we propose that the RBC-MNE frequency could be the result of increased genomic instability due to the presence of all these factors in the same individual. Importantly, this increase in RBC-MNE could be monitored and serve as a biomarker to identify individuals susceptible to genomic instability.

Another possible contributing factor to the instability of the DNA was the appearance of the mutation that generated polydactyly, which was a mutation widely described in cats and could imply that a greater genomic instability be existed, which promoted cancer progression.

### The Micronucleus Test for Vulnerable Organism Detection

Genomic stability is fundamental for cellular homeostasis and the health of an organism; however, during everyday life, all organisms are exposed to endless endogenous and exogenous genotoxic agents which frequently alter genomic homeostasis. Regularly, the damage caused by genotoxins are silent and pass unnoticed. However, these toxins are more evident in diseases characterized by progressive deterioration of specific tissues, cancer susceptibility, chromosome rearrangement, and hypersensitivity to genotoxic agents. Furthermore, genotoxic agents affect individuals in different ways (genetic instability, mutagenesis, teratogenicity, or carcinogenesis), depending on the genetics factor, environment, or disease. This was the main reason for having basic and relatively inexpensive techniques that allowed us to identify the most vulnerable organisms quickly and efficiently with easy-to-follow biomarkers, such as RBC- MNE. Thus, the identification of genetic changes that cause disease included chromosome instability and were important tools for the basis of diagnostic criteria that contributed to a better understanding of the etiology of disease and facilitated decision-making regarding treatment. The genetic changes appeared in the early stages of disease, even earlier than the clinical manifestations, and they could be very useful biomarkers for determining a prognosis (1, 3, 27).

## Conclusion

This was the first case report that described genetic instability determined by the RBC- MNE assay in a cat with hypopigmentation, polydactyly, and squamous cell carcinoma. This report showed that the frequency of RBC-MNE observed in this cat was significantly higher than previously described in domestic, wild, or experimental felines. Due to the high sensitivity of felines to genotoxic damage. Due to the high sensitivity of felines to genotoxic damage, we suggest applying RBC- MNE assay to a greater number of organisms affected with cancer to confirm these results and to establish the use of RBC-MNE as an additional test in clinical practice to detect animals that were vulnerable to genomic instability and this information could contribute to the prognosis and identification of disease conditions in the veterinary patient.

## Data Availability Statement

The raw data supporting the conclusions of this article will be made available by the authors, without undue reservation, to any qualified researcher.

## Ethics Statement

The authors followed the ethical recommendations proposed in clause “For studies that use animals that are owned by the client, high level (best practices) of veterinarians was demonstrated, and written informed consent was received by the client” of the document: ANIMAL USE CONSENT GUIDE of the International Association of Veterinary Editors.

## Author Contributions

MA-G led the generation, analysis and interpretation of the data, drafted the main arguments and developed the first and final version of the full article. MI-P and LM-N collaborated in the generation, analysis and interpretation of the data, and in the first version of the complete article. RL-V-G collaborated in analysis and interpretation of the data, and in the first and final version of the complete article. IC-Y provided the original laboratory data and participate in the analysis and interpretation of first version draft. OT-B provided the original idea to develop the study, directed the analysis of the information and writing the text of the first and final version.

## Conflict of Interest

The authors declare that the research was conducted in the absence of any commercial or financial relationships that could be construed as a potential conflict of interest.
